# Accuracy and Reliability of Multimodal Imaging in Diagnosing Knee Sports Injuries

**DOI:** 10.2174/0115734056360665250506115221

**Published:** 2025-05-15

**Authors:** Di Zhu, Zitong Zhang, Wenji Li

**Affiliations:** 1 Sports College, Shandong Normal University, Jinan 250000, Shandong, China; £ Present Address: Sports College, Shinhan University, Gyeonggi-do 11626, South Korea; 2 Sports College, Shinhan University, Uijeongbu, Gyeonggi-do 11626, South Korea

**Keywords:** Medical image, Sports injuries of knee joints, Deep learning, Convolutional neural networks, Magnetic resonance imaging, Computed tomography, Ultrasound, Magnetic resonance imaging

## Abstract

**Background:**

Due to differences in subjective experience and professional level among doctors, as well as inconsistent diagnostic criteria, there are issues with the accuracy and reliability of single imaging diagnosis results for knee joint injuries.

**Objective:**

To address these issues, magnetic resonance imaging (MRI), computed tomography (CT) and ultrasound (US) are adopted in this article for ensemble learning, and deep learning (DL) is combined for automatic analysis.

**Methods:**

By steps such as image enhancement, noise elimination, and tissue segmentation, the quality of image data is improved, and then convolutional neural networks (CNN) are used to automatically identify and classify injury types. The experimental results show that the DL model exhibits high sensitivity and specificity in the diagnosis of different types of injuries, such as anterior cruciate ligament tear, meniscus injury, cartilage injury, and fracture.

**Results:**

The diagnostic accuracy of anterior cruciate ligament tear exceeds 90%, and the highest diagnostic accuracy of cartilage injury reaches 95.80%. In addition, compared with traditional manual image interpretation, the DL model has significant advantages in time efficiency, with a significant reduction in average interpretation time per case. The diagnostic consistency experiment shows that the DL model has high consistency with doctors’ diagnosis results, with an overall error rate of less than 2%.

**Conclusion:**

The model has high accuracy and strong generalization ability when dealing with different types of joint injuries. These data indicate that combining multiple imaging technologies and the DL algorithm can effectively improve the accuracy and efficiency of diagnosing sports injuries of knee joints.

## 1 INTRODUCTION

Knee joint injury is a common problem in sports medicine. In high-intensity sports, the incidence rate of knee joint injury is high. The anatomical structure of the knee joint is complex, leading to more severe injuries. Precise diagnosis of knee joint injuries is the key to ensuring treatment effectiveness. At present, various imaging technologies have varying sensitivity and specificity for different types of knee joint injuries, and there are differences in the imaging diagnostic criteria for knee joint injuries among different medical institutions and doctors. On this basis, the interpretation of medical images relies on the experience and professional level of doctors, which can easily lead to subjective bias. These issues result in insufficient accuracy and reliability for doctors in using medical images to diagnose sports injuries of knee joints.

To address these challenges, a strategy is researched and proposed. This strategy aims to fully utilize the advantages of high-resolution magnetic resonance imaging (MRI), computed tomography (CT), and ultrasound (US) imaging in soft tissue and skeletal structure imaging to obtain more comprehensive information on knee joint injuries. In this article, deep learning (DL) technology is applied, and convolutional neural networks (CNN) are used for the automatic analysis and interpretation of medical image data. This article aims to improve the accuracy and efficiency of diagnosis by training the model to identify and classify different injury patterns.

This article first outlines the clinical importance of sports injuries of knee joints and the limitations of existing diagnostic methods in the introduction section. Then, in the related work section, the application of various imaging technologies in the diagnosis of knee joint injuries is reviewed, and the potential of the DL algorithm in improving diagnostic accuracy is discussed. Subsequently, the specific steps and implementation details of medical image acquisition, image data processing, automatic analysis, and application of technologies are introduced in detail in the materials and methods section. In the section on evaluating diagnostic technology, the effectiveness of the proposed method is assessed through sensitivity and specificity analysis, time efficiency testing, diagnostic consistency experiments, and evaluation of the model’s generalization ability. Finally, the reference section lists the relevant literature on which this article is based.

This article explores a new method for comprehensive evaluation of sports injuries of knee joints by integrating multiple medical imaging technologies, which improves the accuracy of injury diagnosis.

The use of DL models for automatic analysis of image data reduces reliance on subjective judgments from doctors and enhances diagnostic consistency and reliability.

The advantages of the proposed method in improving diagnostic efficiency, reducing subjective bias, and model generalization ability are verified through experiments, providing an effective auxiliary diagnostic tool for the field of sports medicine.

## 2 RELATED WORK

To improve the accuracy of diagnosis, many researchers have attempted to use different methods to optimize the diagnosis of sports injuries of knee joints. Some studies have shown that although MRI is the preferred method for evaluating knee joint injuries, its diagnostic accuracy has limitations. Meanwhile, several studies have found that ultrasound has high accuracy and low cost in diagnosing knee joint lesions [1-3]. Ni *et al.* [4] explored the application of artificial intelligence-assisted compressed sensing in knee joint MRI scanning and found its potential to improve scanning speed and image quality. Kong AP. *et al.* [5] believed that preliminary imaging assessment of knee joint lesions should start from X-ray anteroposterior and lateral views to identify most knee joint problems. Makaram *et al.* [6] reviewed the literature on the diagnosis and treatment of multi-ligament injuries in adult knee joints. He found that although the number of studies increased over time, most of them had low levels of evidence and lacked diversity, and there was heterogeneity in the result reports. The studies consistently focused on clinical evaluation, imaging, surgical strategy, timing, and rehabilitation. The study of Duong *et al.* [7] emphasized the three common causes of knee joint pain: osteoarthrosis, patellofemoral pain, and meniscus tear. These diseases could be diagnosed clinically through symptoms and physical examination. Although these diseases might lead to significant disabilities, first-line treatment typically focuses on non-surgical conservative management, such as exercise therapy, education, and self-management, to help patients effectively control their condition. For specific situations, surgical treatment might be necessary. Helito *et al.* [8] retrospectively analyzed MRI scans and clinical instability tests of 95 patients with anterior cruciate ligament tears and found that abnormalities in the anterolateral ligament were significantly correlated with pivot shift test results. The MRI abnormalities of the anterolateral ligament were the only significant variable for predicting the outcome of high pivot shift, indicating that the abnormalities of the anterolateral ligament had important value in the diagnosis of sports injuries of knee joints. Rakhra [9]. analyzed preoperative MRI and surgical results of 39 patients with acute dislocation of the knee joint and found that MRI had high accuracy in diagnosing posterolateral corner structure tears, especially injuries to the lateral collateral ligament, popliteus tendon, biceps femoris muscle tendon, and ligament-capsular complex. However, there were differences in the correlation between MRI and surgery in terms of structural integrity grading, suggesting that clinical doctors should combine MRI results with intraoperative exploration when evaluating the stability of knee joint dislocation. Ochi *et al.* [10] pointed out that subchondral insufficiency fracture of the knee (SIFK) was a common cause of knee pain in elderly people. MRI was the key to early diagnosis of SIFK. Differential diagnosis included other osteochondral lesions, and attention should be paid to the association between cartilage lesions and meniscus abnormalities with SIFK. Although MRI and ultrasound play an important role in the diagnosis of knee joint injuries, their accuracy and application scope still have limitations, and imaging methods need to be further optimized to improve diagnostic precision.

Some researchers have attempted to improve diagnostic consistency and accuracy by using other methods to address these issues. Researchers used the DL algorithm to establish a model for detecting anterior cruciate ligament injuries in the knee joint, and there was no significant difference between the model diagnosis and doctors’ diagnoses [11]. Tran *et al.* also used DL for diagnosis. They achieved high accuracy through training on nearly 20000 MRI images and validated their generalization ability on external datasets in different countries, significantly improving the diagnostic efficiency of sports injuries of knee joints [12]. Kim *et al.* [13] developed an integrated DL model for predicting anterior cruciate ligament tears of the knee joint from lateral knee X-rays and evaluated its diagnostic performance. The model was trained and tested on 1433 X-rays from two medical centers, and the results showed that its sensitivity, specificity, and accuracy were comparable to those of musculoskeletal radiologists. Santomartino *et al.* [14] reviewed and analyzed 19 studies. They found that the DL model demonstrated high accuracy in MRI detection of knee joint ligament and meniscus tears, significantly improving the diagnostic efficiency and accuracy of sports injuries of knee joints, which indicated broad application prospects in this field in the future. Houserman *et al.* [15] developed a model for predicting whether patients were suitable for total knee arthroplasty, single-chamber knee arthroplasty, or surgery-free by analyzing 3-view X-ray images of 2767 patients, and the results showed high accuracy in predicting indications for knee arthroplasty surgery. Hinterwimmer *et al.* [16] reviewed the application of machine learning in knee arthroplasty surgery and found that machine learning models could effectively predict complications, costs, functional outcomes, *etc*., but the accuracy of predicting more complex outcomes needed to be improved. Their study emphasized the importance of specific data input and interdisciplinary collaboration, providing direction for the improvement of diagnostic methods for sports injuries of knee joints in the future. Regarding the issue of diagnostic accuracy, researchers have significantly improved the diagnostic efficiency and accuracy of sports injuries of knee joints by applying technologies such as DL, demonstrating broad application prospects.

## 3 MATERIALS AND METHODS

### 3.1 Medical Image Acquisition

Precise medical image acquisition is the key foundation for subsequent analysis and treatment decision-making in the diagnosis of sports injuries of knee joints. To ensure a comprehensive evaluation of the bone, cartilage, ligaments, meniscus and other structures of the knee joint, this article integrates and applies multiple imaging technologies. Imaging technologies include MRI, CT, and US, each with its own advantages based on its imaging characteristics, indications, and imaging effects on different tissues to ensure comprehensive and precise injury detection. MRI images are from the MRNet dataset, which collects 1,370 knee MRI images; CT images are from the Emory Knee Radiograph Dataset, which uses 10,000 images; US images are from the knee injury detection dataset, which uses 7,000 images. The dataset contains a total of about 18,370 images. In data processing, the image data is divided into a training set (accounting for 80%) and a validation set (accounting for 20%). In order to improve the generalization ability and robustness of the model, data enhancement techniques are used, including random cropping, rotation, and flipping operations. All images are uniformly adjusted to 256×256 pixels after preprocessing to meet the requirements of model input.

MRI has high-resolution imaging capabilities for soft tissues and is the preferred tool for doctors to evaluate structural injuries such as ligaments, meniscus, and cartilage. During MRI scanning, the patient is in a supine position, and the knee joint is placed in a specific coil device to minimize motion artifacts and ensure image quality. Based on the preliminary clinical assessment of knee joint injury, imaging is performed in the coronal, sagittal, and axial planes to clearly demonstrate joint structure from multiple angles. MRI uses multiple imaging sequences to enhance visualization of different knee joint structures. Longitudinal relaxation time-weighted imaging is used to provide clear anatomical structure information, and transverse relaxation time weighting and proton density weighting are used to identify edema, bleeding, and other pathological changes within tissues. Fat suppression technology can effectively eliminate fat signals and further improve the contrast of soft tissue injury. Fig. (**1**) shows MRI images of different imaging sequences of the knee joint:

To accurately capture the subtle structures inside the knee joint, thin-slice scanning of 1 millimeter is used. Thin-slice scanning can more precisely display small tears, fissures, and injury sites in knee joints, and has significant advantages in identifying meniscus tears and early cartilage injuries.

CT is mainly used in the diagnosis of sports injuries of knee joints to evaluate bony structures. When there are suspected fractures, osteochondral injuries, or irregular joint surfaces, CT has greater advantages. The data collected by CT can be post-processed using multi-plane reconstruction technologies to reconstruct axial images into coronal, sagittal, and other planes, providing a more comprehensive view of the joint surface. For complex knee joint fractures or osteochondral injuries, 3D imaging technology is used to further enhance the visualization of fracture lines, fragment positions, and irregular joint surface shapes. CT is not as good as MRI in soft tissue imaging, but dual-energy CT technology can better distinguish different types of tissues by using X-rays of different energy levels. This technology has a high sensitivity for evaluating pathological changes and osteochondral injuries in the subchondral bone of the knee joint. Especially in cases where MRI is contraindicated or impossible, dual-energy CT can replace MRI. The two technologies complement each other and are chosen based on the actual situation of the patient. Fig (**2**) shows CT images of the knee joint:

Fig. (**2**) shows CT images of the knee joint above the axial plane. Compared to MRI images, CT images have higher resolution in displaying skeletal structures.

US imaging plays a significant role in the assessment of injuries to superficial structures of tendons and ligaments. High-frequency probes are used in US imaging to image superficial tissues. To improve the display of lesions, US imaging enhances hemodynamics information by using color Doppler technology. This makes it easier to identify changes in blood flow during inflammation or acute injury. Unlike MRI and CT, ultrasound can evaluate soft tissue injuries of the knee joint under different movement states through dynamic imaging. During flexion, extension, and weight-bearing movements in the knee joint, dynamic changes in tendons and ligaments can be observed through US imaging. US imaging plays a role in the dynamic evaluation of sports injuries such as patellar tendinitis and hamstring muscle injuries. Fig. (**3**) shows the US images of the knee joints.

In Fig. (**3**), the left image is a US image of a normal left knee, and the right image is a US image of inflammation in the right knee joint. US imaging and color Doppler technology help doctors accurately assess injuries and inflammation.

Combining MRI, CT, and US technologies to comprehensively evaluate knee joint injuries can improve diagnostic accuracy and reliability. These technologies complement each other, promptly detecting various types of tissue injury and providing a foundation for subsequent analysis and diagnosis.

### 3.2 Image Data Processing

After medical image acquisition, image enhancement, noise elimination, and tissue segmentation are performed on it. By precisely identifying and extracting different structures of the knee joints, high-quality input data can be provided, thereby improving the accuracy and reliability of diagnosis.

The original acquisition of medical images is limited by factors such as device resolution, patient movement, and environmental noise, resulting in poor imaging quality. This article uses histogram equalization [17, 18] to enhance the collected medical imaging images. Histogram equalization adjusts the gray level distribution of the image, expands the contrast range of the images, and enhances the contrast of soft tissue structures such as cartilage, meniscus, and ligaments. The formula for histogram equalization is:


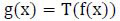


In Formula (1), f(X) is the grayscale value of MRI, CT, and US medical images; T is the equalization transformation function; g(X) is the grayscale value of the output medical image. Histogram equalization is crucial for the precise detection of soft tissue injuries in the knee joint, as it can effectively enhance the clarity of the boundaries between soft tissue and surrounding tissues. It can reduce the omission of subtle injured areas caused by low contrast and improve the recognition rate of early lesions.

In response to noise interference, especially the common Gaussian noise in MRI images, this article uses Gaussian filtering [19, 20] to suppress noise while preserving the edge details of the image. Gaussian filtering is suitable for situations where noise is uniformly distributed, and the following formula is used:


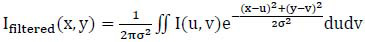


In Formula (2), I_filtered_(x, y) is the pixel value of the filtered medical image; I(u, v) is the pixel value of the original medical image; σ is the standard deviation of the Gaussian function, which determines the smoothness of the filtering. Fig. (**4**) shows the image processing effects after image enhancement and noise elimination.

In Fig. (**4**), from top to bottom are the original images, the Gaussian-filtered images, and the histogram equalization-processed images. The left side of the original images is MRI images of knee joints, and the right side is CT images of knee joints. By using Gaussian filtering and histogram equalization, image quality can be significantly improved. Details can be enhanced, and noise can be reduced, thereby providing clearer diagnostic criteria.

The anatomical structure of the knee joint is complex, with subtle boundaries between different tissues. In this article, the U-Net [21, 22] network structure is used to segment knee joint tissue. U-Net can be used to segment tissues such as bones, ligaments, meniscus, and cartilage in MRI and CT images. U-Net has a skip connection mechanism that combines low-resolution spatial information from the encoder with high-level semantic information from the decoder, improving its ability to recognize complex anatomical structures of the knee joint. This method has a significant segmentation effect on small lesions. The segmentation optimization of U-Net is achieved by minimizing the cross-entropy loss function L.


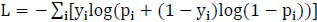


Taking a meniscus tear in the knee joint as an example, y_i_ represents the probability that a pixel on a medical image belongs to a meniscus tear, indicating whether the point is manually labeled or known to belong to a meniscus tear. Its value is 0 or 1. p_i_ is the probability predicted by the U-Net model that the pixel is a torn area with a value between 0 and 1.

### 3.3 Automatic Analysis

In the imaging diagnosis of sports injuries of knee joints, this article uses DL technology as the core to automatically analyze and classify different types of knee joint injuries from complex imaging data. The processed MRI, CT, and US images are labeled for model training.

To construct and train the model, this article chooses CNN [23, 24] as the core algorithm. CNN is one of the most widely used algorithms for image processing in DL. In knee joint image analysis, CNN can automatically identify different tissue structures and injury areas.

In the analysis of knee joint imaging, the anatomical structure of the knee joint is complex, and the tissue types are diverse, including bones, cartilage, ligaments, *etc*. For these complex organizational structures, models are first constructed for the automatic extraction of local features in the images. The convolutional layer captures local information in the images by learning different convolutional kernels, including the continuity of bone edges, the integrity of cartilage, and the subtle structures of ligament rupture or tear. Through the operation of convolutional layers, the DL model learns the spatial distribution characteristics of images and automatically detects the boundaries and injury features of different tissues within the knee joint without relying on manual feature engineering.

This paper adopts a stacked multi-layer convolution structure when designing the model. The model contains 5 convolution layers, and each convolution layer is followed by a pooling layer to gradually extract image features and reduce the spatial dimension of the feature map. The convolution kernel size of the convolution layer is 3×3, the step size is 1, and the padding method is “same” to maintain the size of the feature map. The pooling layer uses maximum pooling, and the pooling window size is 2×2. After 5 convolution and pooling operations, the feature map is flattened and input to the fully connected layer. The model contains 2 fully connected layers. The first fully connected layer has 1024 neurons, and the second fully connected layer outputs the final classification result. The activation function uses ReLU to introduce nonlinear features. When training the model, the learning rate is set to 0.001, the optimizer uses the Adam algorithm [25, 26], and the Epoch value is set to 50 to improve the accuracy of injury recognition.

Due to the high cost of labeling knee joint MRI, CT images, and US images, obtaining a large amount of high-quality labeled data is extremely difficult. To address this issue, this article applies transfer learning technology in training [27, 28]. The core idea of transfer learning is to use pre-trained DL models on large medical imaging datasets as the basis for fine-tuning specific tasks. A pre-trained model using ImageNet is fine-tuned based on collected medical images for specific types of knee joint injuries. This process retrains the high-level neurons of the model while keeping the weights of low-level neurons unchanged, allowing the model to adapt further to specific features of knee joint images while retaining its general feature extraction ability.

Transfer learning reduces reliance on large-scale knee joint imaging data. The model is fine-tuned on a small dataset, and high diagnostic precision can still be achieved. For the fine classification of anterior and posterior cruciate ligaments and meniscus, transfer learning can maintain high classification accuracy with a small amount of labeled data.

The complexity of knee joint injury types and the differences in tissue features captured by different imaging technologies make it difficult for a single classifier to comprehensively cover all types and features of injuries. This article uses ensemble learning [29, 30] and multi-task learning [31, 32] to improve the detection and classification precision of knee joint injuries. In this study, the Bagging algorithm was used as a meta-learner for ensemble learning to enhance diagnostic performance by integrating the prediction results of multiple CNN models. The Bagging algorithm generates multiple sub-datasets by random sampling and independently trains a CNN model on each sub-dataset. The final prediction result is a fusion of the outputs of each model by weighted averaging. The hyperparameters are set as follows: 10 base learners are configured, each model is trained on an independent sub-dataset; 50% of each sub-dataset is randomly sampled, and repeated sampling is allowed. The process of applying two methods is shown in Fig. (**5**).

Fig. (**5**) shows the application process of ensemble learning and multi-task learning. For MRI, CT, and US images, the DL model performs specialized feature extraction for different images. MRI has a high resolution in detecting ligament tears and meniscus injuries. CT has stronger imaging capabilities for fractures and bone wear. The US is suitable for real-time observation of the movement states of knee joint soft tissues and small-scale minor injuries. Multiple DL models are trained for MRI, CT, and US to extract features from different images. Bagging technology is used to fuse the classification results of each model through weighted averaging. Through Boosting technology, the weights and structure of the model are gradually adjusted for some injury types that are difficult to identify, such as tearing and hidden injuries, in order to enhance sensitivity to these injuries.

In the process of using MRI, CT, and US imaging data together for knee joint injury diagnosis, multi-task learning can help to simultaneously process detection tasks for multiple types of injuries, thereby improving the efficiency and accuracy of the model. Multi-task learning designs multiple task output heads in network architecture, with each output head responsible for a specific injury classification task. One output head focuses on the classification of meniscus tears and is responsible for detecting anterior cruciate ligament tears. Another output head identifies cartilage and bone injuries. According to the actual diagnostic requirements, the number of task output heads can be adjusted. These output heads share the feature extraction module of the underlying network, enabling the model to extract universal and specific features from MRI, CT, and US images.

### 3.4 Application of Technologies

The combination of imaging technology and DL provides precise and reliable support for early diagnosis, injury classification, and rehabilitation monitoring in the diagnosis and treatment of knee joint injuries in athletes. This article discusses its application effect through standardized image interpretation and dynamic follow-up and monitoring. Rapid and accurate diagnosis is crucial in determining treatment plans for knee joint injuries in athletes. The standardized image interpretation process is combined with MRI and other imaging technologies, as well as DL automatic analysis. Fig. (**6**) shows the diagnosis and rehabilitation process of knee joint injury:

As shown in Fig. (**6**), after image acquisition, image enhancement technology is used to reduce noise in the image and enhance important anatomical structures. Automatic analysis uses CNN to detect and classify injury areas in images. CNN automatically labels the injury area and generates a diagnostic report, and it automatically measures the injury area quantitatively based on standardized algorithms and uses clear criteria to classify the severity of the injury. This standardized interpretation process can ensure diagnostic consistency among institutions, reduce errors in human interpretation, and provide objective references for clinical doctors. Due to the application of DL and standardized processes, different institutions are able to maintain a high degree of consistency in diagnosis results. Doctors from different hospitals can share standardized knee joint imaging data and automatic analysis results through the cloud, reducing diagnostic bias caused by regional and equipment differences.

The rehabilitation of knee joint injuries is a long-term process, and dynamic monitoring of the healing of the injury is crucial for adjusting the rehabilitation plan. During the rehabilitation process, patients need to undergo regular imaging examinations of the knee joint to monitor the progress of healing at the site of injury. At each follow-up, the latest images of the knee joint are obtained through imaging technologies such as MRI or CT. CNN compares these new images with the images at the initial diagnosis to quantify the healing progress of the injury. For patients with meniscus tears, CNN can quantitatively evaluate the healing status of the meniscus tear area through multiple imaging analyses, including the recovery length of the tear edge and the degree of cartilage regeneration. If CNN analysis shows slow recovery of tear edges, doctors may consider extending conservative therapy time or changing the intensity of rehabilitation training.

Based on dynamic follow-up data and combined with CNN analysis results, doctors can adjust rehabilitation treatment plans in real-time. Among some patients, CNN analysis may indicate inflammation or joint dysfunction during the healing process, and doctors can take timely intervention measures based on this to avoid worsening of the condition.

The remote monitoring system combining DL and cloud technology enables patients to receive dynamic image analysis at any time during the rehabilitation process without the need to frequently visit the hospital. Patients can undergo regular imaging scans, and the data is automatically uploaded to the cloud for analysis by CNN, generating rehabilitation reports for doctors’ reference.

The application of standardized image interpretation and dynamic follow-up monitoring can improve the diagnostic accuracy and treatment effectiveness of knee joint injuries. Standardized interpretation reduces subjective differences among doctors and ensures the uniformity of diagnosis results. Meanwhile, the utilization of dynamic monitoring systems makes the rehabilitation process more scientific and personalized.

## 4 RESULTS AND DISCUSSION

### 4.1 Sensitivity and Specificity Testing

In order to evaluate the diagnostic accuracy and reliability of deep learning models based on MRI, CT and ultrasound (US) imaging technology for knee sports injuries, the sensitivity and specificity of the model were tested in the experiment. A total of 2100 image data were collected in this study, covering three modalities: MRI, CT and ultrasound. These image data came from the imaging department of a hospital in a certain city, spanning from January 2022 to December 2023. The dataset includes a variety of knee injury types, and this experiment focuses on four main types of injuries: anterior cruciate ligament tear, meniscus injury, cartilage injury and fracture. The image data were randomly shuffled into three groups, and the data were input into the deep learning model for automatic analysis and classification to predict the type of injury for each patient. The model's prediction results were compared with the actual diagnosis (the results marked by the doctor), and the model's sensitivity and specificity were calculated to measure the model's diagnostic effect on different knee injuries. Sensitivity refers to the proportion of injured areas correctly identified by the model, and specificity refers to the proportion of uninjured areas correctly identified by the model. Model performance evaluation includes prediction accuracy. The statistical experimental results are shown in Table **1**.

Table **1** shows the diagnostic performance of the DL model for four types of knee joint injuries, including sensitivity, specificity, and diagnostic accuracy. In the diagnosis results of anterior cruciate ligament tear, the sensitivity ranges from 87.45% to 91.25%; the highest specificity is 86.55%; the diagnostic accuracy data is above 90%. In the diagnosis of cartilage injury, the highest diagnostic accuracy reaches 95.80%, which is also the highest diagnostic accuracy in the entire experiment. The sensitivity of fracture diagnosis is the highest at 90.41%, and the diagnostic accuracy is the highest at 93.50%. The experimental results indicate that the DL model has high sensitivity and specificity in diagnosing sports injuries of knee joints, but different types of injuries and sample sizes can affect the diagnostic accuracy of the model. The small total number of diagnoses used for testing in this experiment is also one of the reasons for the differences in data.

### 4.2 Time Efficiency Testing

This experiment aims to compare the time efficiency of traditional manual image interpretation and deep learning models in the diagnosis of knee sports injuries, verify the advantages of automatic analysis in this paper and does not require a large sample size to verify the accuracy of the model. Manual interpretation requires a lot of time and manpower, so choosing a smaller sample size can effectively control the cost of the experiment while ensuring the feasibility and efficiency of the experiment. To this end, this experiment took out a total of 250 images of knee injury cases from the imaging data, mainly considering five types of injuries: anterior cruciate ligament tear, meniscus injury, patellar cartilage injury, fracture and medial collateral ligament injury. These types of injuries are most common in clinical practice and have a significant impact on patients' motor ability and rehabilitation process. The manual group consisted of 3 experienced radiologists who independently interpreted the images and recorded the diagnosis time, and recorded the time consumed by the two methods to complete the same amount of image processing. The results of the five types of injuries are shown in Table **2**.

The data in Table **2** shows a comparison of time efficiency between the traditional manual interpretation group and the DL model group in the imaging diagnosis of different types of knee joint injuries. The average interpretation time per case for the five types of injuries in the manual group is the lowest at 17.8 minutes, with a total interpretation time ranging from 8.1 to 17.2 hours. In contrast, the average interpretation time per case in the DL model group is significantly reduced, with the lowest average interpretation time per case being only 4.4 minutes and the longest total interpretation time being 5 hours. The reason for the time difference is that manual interpretation relies on doctors’ individual evaluations, while the DL model reduces the time consumption caused by manual operations through automatic analysis. The experimental results show that the DL model has significant advantages in diagnostic efficiency.

### 4.3 Diagnostic Consistency Experiment

The diagnostic consistency experiment aims to evaluate the consistency between the DL model’s automatic diagnosis of knee joint injuries and the doctors’ manual interpretation, especially in reducing subjective bias. Fifty patients with sports injuries of knee joints are selected for the experiment, and each patient’s imaging data is independently interpreted by three doctors. The DL model is used to automatically analyze the same imaging data. The consistency of interpretation among doctors and between doctors and the DL model is compared using Cohen’s Kappa coefficient. Cohen's Kappa coefficient is used to measure the consistency of classification results, taking into account the influence of random consistency. It quantifies the degree of consistency beyond the random level by comparing the difference between the actual observed consistency ratio and the random consistency ratio. Kappa values range from -1 to 1, where 1 indicates perfect consis-tency, 0 indicates the same as random consistency, and negative values indicate that the consistency is lower than the random level. The error rate of the DL model relative to the doctors’ diagnosis results is calculated. The error rate is calculated as the percentage of the error in the size of the lesion in the diagnosis results. The results obtained are shown in Fig. (**7**).

Fig. (**7**) shows the diagnostic consistency test results of 50 patients with sports injuries of knee joints, including consistency among doctors, consistency between doctors and the DL model, and error rate of the DL model. The data results show a high level of consistency among doctors, and overall, it is higher than the consistency between the DL model and doctors. The consistency between doctors and DL models is lower than that among doctors, but the lowest value is still 0.86, indicating good performance. High consistency among doctors means that their diagnoses are already relatively consistent, but there is still subjectivity in the standards and judgments of different doctors. If the DL model can be consistent with the doctors’ diagnoses, it suggests that the model effectively reduces these subjective differences. The overall error rate of the DL model is below 2%, indicating a relatively small classification error. The experimental results show that the DL model has high consistency and small errors in the automatic diagnosis results of knee joint injuries with the interpretation of doctors.

### 4.4 Model Generalization Ability Testing

To test the generalization ability of the DL model in the diagnosis of other types of joint injuries, this experiment applies the model to medical image analysis of the shoulder joint, ankle joint, elbow joint, and hip joint. Medical images of the shoulder, ankle, elbow, and hip joints are collected, and each type of data is labeled with the type of injury to train the model. After training, three experiments are conducted on each of the four types of joint injuries on the new dataset, with different sample sizes for each experiment. The diagnostic accuracy of the model is calculated, and the results obtained are shown in Fig. (**8**).

Fig. (**8**) shows the image situation of different joint types, and each subgraph displays the sample size and accuracy of three experiments. The diagnostic accuracy of shoulder joint injury reaches a maximum of 92.3%, which is the highest value among all groups in this experiment. The accuracy is the lowest in the third experiment of ankle joint injury, at 85.9%. Overall, different types of joint injuries exhibit certain differences in sample size and accuracy. The accuracy of shoulder joint injuries is high due to the more obvious characteristics of the shoulder joint, and the model can better classify them. The accuracy of the third ankle joint experiment is relatively low due to the small number of test samples. The experimental results demonstrate the high accuracy of the model in handling different types of joint injuries, demonstrating its strong generalization ability.

## CONCLUSION

This article significantly improves the accuracy and reliability of diagnosing sports injuries of knee joints by comprehensively using multi-modal imaging technologies such as MRI, CT, and US combined with DL. This article not only demonstrates the efficiency and consistency of the deep learning model in identifying various types of knee joint injuries but also verifies the significant advantages of the model in terms of time efficiency compared to traditional methods. In addition, experimental evaluation confirms that the model has good generalization ability and can adapt to the diagnostic needs of different joint injuries. Despite these achievements, future research still needs to further validate the stability and applicability of the model in larger sample sizes and diverse clinical environments. Exploration of more advanced algorithms to further improve the diagnostic precision of the model and how to better integrate these technologies into clinical workflows are important directions in future research. Considering cost-effectiveness and practical feasibility, future work should also focus on how to optimize algorithms to adapt to resource-limited medical environments. In addition, the interpretability of the model remains an important research direction. Deep learning models are often regarded as “black boxes”, which to some extent limits their widespread application in clinical practice. Future research will explore the use of visualization techniques to highlight the key feature areas identified by the model and develop interpretation tools to help clinicians understand the diagnostic logic of the model.

## Figures and Tables

**Fig. (1):**
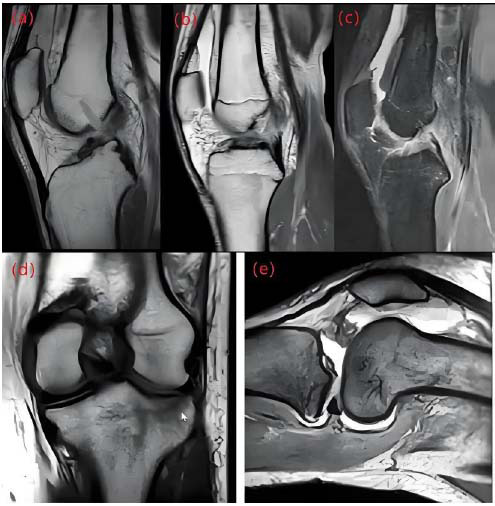
MRI images of knee joint. (**a**, **b**, and **c**) show the images of longitudinal relaxation time-weighted, transverse relaxation time-weighted, and proton density-weighted knee joints, respectively. **(d** and **e**) show knee joint images weighted by coronal longitudinal relaxation time and sagittal transverse relaxation time. Through these multi-angle and multi-sequence MRI images, doctors can gain a more comprehensive understanding of the pathological status of the knee joint

**Fig. (2):**
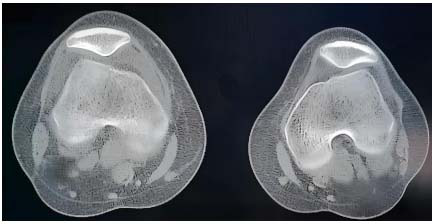
CT of the knee joint.

**Fig. (3):**
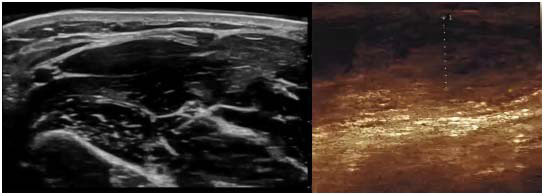
US images of knee joints.

**Fig. (4):**
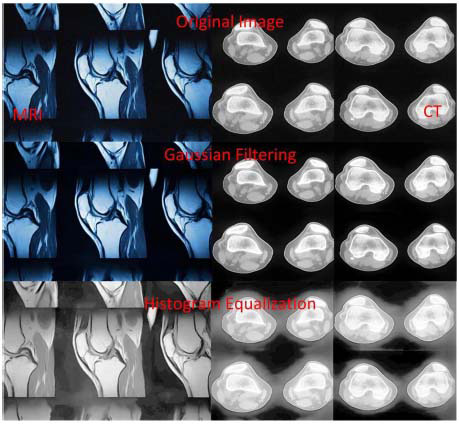
Image processing effects after image enhancement and noise elimination.

**Fig. (5):**
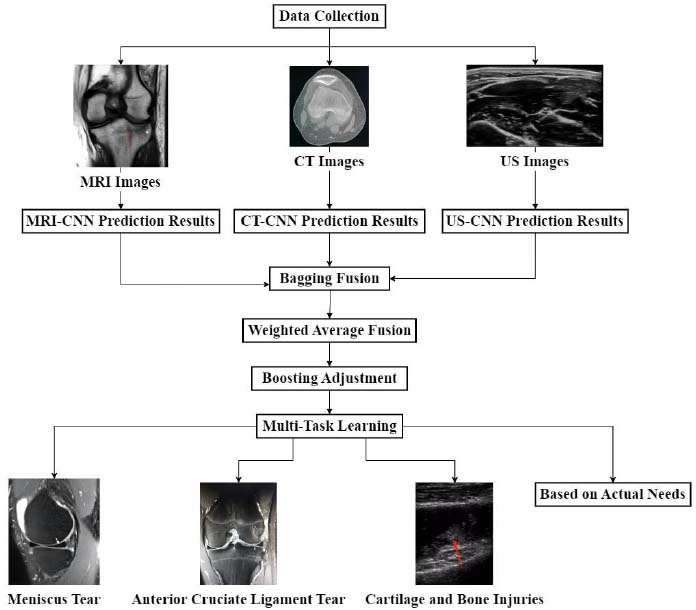
Application process of ensemble learning and multi-task learning.

**Fig. (6):**
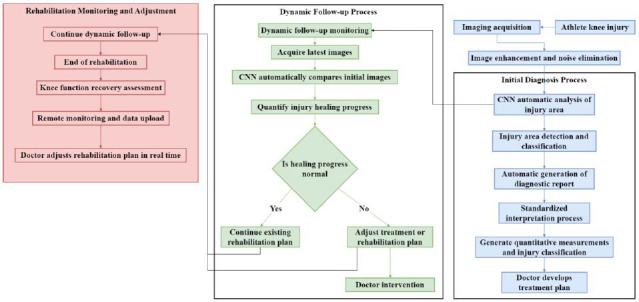
Diagnosis and rehabilitation process of knee joint injury.

**Fig. (7):**
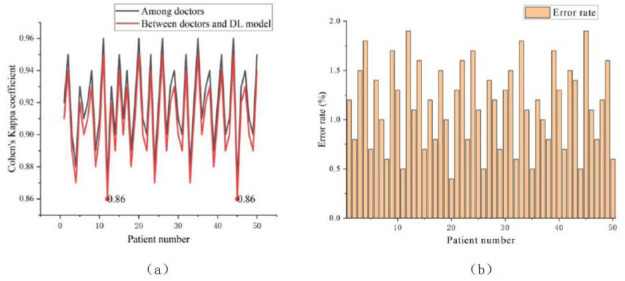
Results of diagnostic consistency and error rate. (**a**) Results of diagnostic consistency. (**b**) Error rate.

**Fig. (8):**
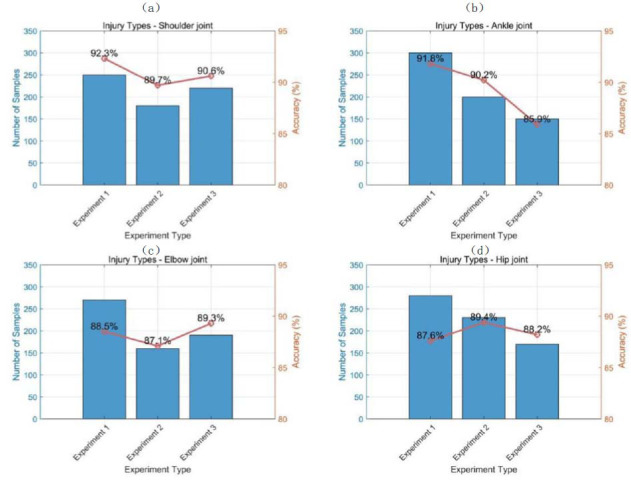
Diagnostic test results of each joint. (**a**) Injury Types - Shoulder joint. (**b**) Injury Types - Ankle joint. (**c**) Injury Types - Elbow joint. (**d**) Injury Types - Hip joint.

**Table 1:** Model test results.

Injury Type	Number of Diagnoses	Sensitivity	Specificity	Diagnostic Accuracy
Anterior cruciate ligament tear	202	91.25%	84.35%	92.72%
	205	87.45%	86.55%	90.85%
	200	90.41%	85.15%	93.53%
Meniscus injury	182	85.82%	85.02%	85.94%
	178	93.13%	87.56%	94.76%
	180	88.26%	85.03%	86.51%
Cartilage injury	152	91.85%	89.42%	94.50%
	149	93.77%	90.21%	95.80%
	150	91.25%	84.35%	92.72%
Fracture	125	87.45%	80.56%	90.85%
	123	90.41%	85.15%	93.50%
	120	85.83%	85.03%	85.91%

**Table 2:** Time efficiency test results.

Injury Type	Number of Diagnoses	Average Image Interpretation Time per Case (min)		Total Interpretation Time (hours)	
		Manual Group	DL Model Group	Manual Group	DL Model Group
Meniscus injury	58	17.8	5.2	17.2	5
Anterior cruciate ligament tear	45	19.3	4.5	14.5	3.4
Patellar cartilage injury	37	18.2	4.9	11.2	3
Medial collateral ligament injury	34	20.1	5	11.4	2.8
Fracture	26	18.7	4.4	8.1	1.9

## Data Availability

Data sharing is not applicable to this article as no new data were created or analyzed in this study.

## LIST OF ABBREVIATIONS

CT: Computed tomography
MRI: Magnetic resonance imaging
US: Ultrasound
DL: Deep learning
CNN: Convolutional neural networks
SIFK: Subchondral insufficiency fracture of the knee
